# Comprehensive multiregional analysis of molecular heterogeneity in bladder cancer

**DOI:** 10.1038/s41598-017-11291-0

**Published:** 2017-09-15

**Authors:** Mathilde Borg Houlberg Thomsen, Iver Nordentoft, Philippe Lamy, Søren Vang, Line Reinert, Christophe Kamungu Mapendano, Søren Høyer, Torben F. Ørntoft, Jørgen Bjerggaard Jensen, Lars Dyrskjøt

**Affiliations:** 1 Department of Molecular Medicine, Aarhus University Hospital, Palle Juul-Jensens Boulevard 99, 8200 Aarhus N, Denmark; 2 Department of Biomedicine, Aarhus University, 8000 Aarhus C, Denmark; 3 Department of Pathology, Aarhus University Hospital, Ndr. Ringgade, 8000 Aarhus C, Denmark; 4 Department of Urology, Aarhus University Hospital, Palle Juul-Jensens Boulevard 99, 8200 Aarhus N, Denmark

## Abstract

Genetic alterations identified in adjacent normal appearing tissue in bladder cancer patients are indicative of a field disease. Here we assessed normal urothelium transformation and intra-tumour heterogeneity (ITH) in four patients with bladder cancer. Exome sequencing identified private acquired mutations in a lymph node metastasis and local recurrences. Deep re-sequencing revealed presence of at least three and four subclones in two patients with multifocal disease, while no demarcation of subclones was identified in the two patients with unifocal disease. Analysis of adjacent normal urothelium showed low frequency mutations in patients with multifocal disease. Expression profiling showed intra-tumour and intra-patient co-existence of basal- and luminal-like tumour regions, and patients with multifocal disease had a greater degree of genomic and transcriptomic ITH, as well as transformation of adjacent normal cells, compared to patients with unifocal disease. Analysis of the adjacent urothelium may pave the way for therapies targeting the field disease.

## Introduction

Bladder cancer is a common malignant disease, and is the cause of 165.000 cancer related deaths annually^1^. When developing personalized medicine, it is important to understand the biological disease basis and to study disease evolution in order to guide treatment decisions and increase survival rates. Disease evolution and subclone development are associated with varying degrees of intra-tumour heterogeneity (ITH) in different cancer types^2–8^, including bladder cancer^9^. For administering optimal and rational targeted therapeutics, it is important to identify genomic alterations that arose early in cancer development, and hence are present in all cancer cells^10^, or e.g. are present in aggressive subclones giving rise to metastatic lesions.

Previous studies have proven that critical subclones may be missed by analysis of bulk tumours^3, 4^, whereas other studies have shown a more modest level of ITH^5^, or varying degree of ITH between patients^2, 8^. Studies in bladder cancer have shown a low level of ITH within single biopsies, but large differences between primary tumours and metastatic lesions^9, 11^. ITH has been largely overlooked in many studies, and this may be the reason why many biomarkers fail clinical validation^12^.

Multifocal and recurrent bladder tumours are hypothesized to originate from a bladder field disease^13, 14^. Studies analysing the adjacent normal appearing urothelium (field disease) have revealed that the urothelium is highly affected by the disease, harbouring genetic alterations shared with the tumours^15–18^. Furthermore, recent studies applying full-genome and exome sequencing have documented a clonal relationship between metachronous tumours, supporting the field disease model^11, 19^. The field disease and clonal relationship between tumours could be caused by intraepithelial migration and/or luminal seeding and implantation of carcinoma cells from existing tumours - eventually giving rise to recurrent tumours. Another explanation could be the transformation of a stem cell embedded in the urothelium followed by clonal expansion^13, 14^.

To investigate the level of ITH in bladder cancer, and to analyse the extend of mutations observed in adjacent normal urothelial cells we performed multi-regional whole exome sequencing (WES). This was followed by deep targeted sequencing of multiple laser micro-dissected (LMD) cells from tumour regions and normal biopsies from four patients with advanced bladder cancer. Simultaneously, we addressed transcriptional heterogeneity in the LMD regions. We identified varying degrees of mutational ITH, which was further reflected at the transcriptomic level in molecular subtypes. Finally, we observed multiple tumour specific mutations at low frequencies in the adjacent normal samples.

## Results

### Whole exome sequencing

DNA was extracted from 17 tumour samples and from leucocytes procured from four patients (patients 1 to 4) with advanced bladder cancer, treated with radical cystectomy and lymphadenectomy. Tumours from patients 1 and 2 were multifocal while tumours from patients 3 and 4 were unifocal. Clinical and histopathological details are listed in Supplementary Table S1. We performed WES of tumour DNA and matched leucocyte DNA as germline and obtained a mean read depth of 65x (31x–113x). We identified 232–755 mutations (categories 1–2; see Methods) in the four patients. Of these, 178–497 mutations were predicted to have a functional impact (Supplementary Table S2, see Methods). Analysis of mutations with a predicted functional impact revealed highly variable degree of ITH, and comparisons to a lymph node metastasis/local recurrences demonstrated acquisition of multiple private mutations and potentially novel disease driver mutations (Supplementary Fig. S1). We identified mutations in known cancer driver genes^20^ and in proposed drivers in bladder cancer^21^. Mutations in driver genes were identified, both as shared between all bulk exomes within a patient (60–95%), and as private being spatially confined to few or single exomes (5–40%).

### Multi-region targeted amplicon sequencing and expression profiling analysis

In order to assess the mutational ITH, we designed two amplicon panels targeting all mutations with a predicted functional impact (n = 1435 and n = 802). These panels were used to sequence DNA procured from multiple LMD regions from the tumour samples initially analysed by WES (n = 129 LMD regions, Supplementary Figs S2–S5), DNA from a lymph node metastasis (n = 1), DNA from local recurrences (n = 2, see Supplementary Table S1), DNA from normal appearing urothelial cells (n = 28), and leucocyte DNA (n = 4). A mean read depth of 3049x (see Supplementary Table 2) was achieved leading to a better assessment of the presence of a mutation and its frequency compared to WES data. For validation purpose the amplicon panels were also used to sequence the bulk samples (n = 17) used for WES. WES identified mutations (categories 1–2) showed a validation rate of 93.3%, while the remaining mutations (category 3) showed a validation rate of 63.7%. Only validated mutations were included in subsequent analyses. Finally, gene expression profiling (Fluidigm) was used to assess basal- and luminal subtypes, cellular differentiation level, and disease aggressiveness^22, 23^.

### Comprehensive analysis of intra tumour heterogeneity in patients 1–4

#### Patient 1

**Figure 1:**
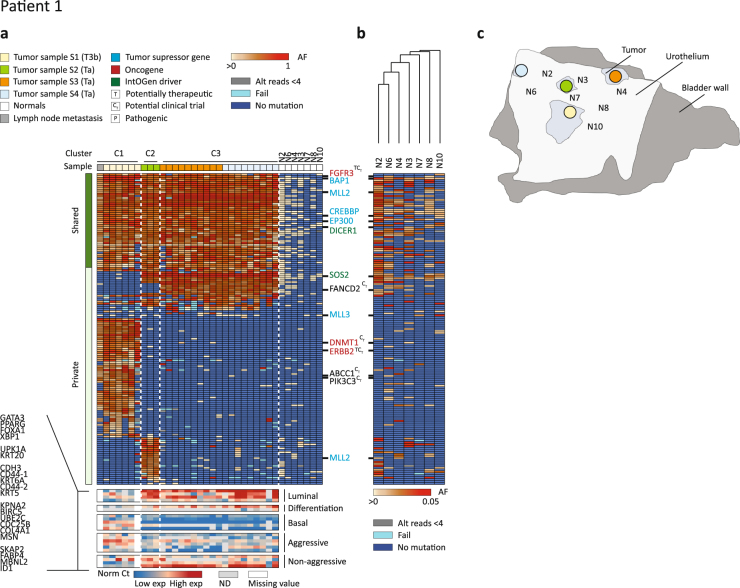
Detailed analysis of patient 1. (**a**) Upper heat map: Deep targeted sequencing was applied to 28 LMD regions from four tumour samples (one from each of the four tumours), a lymph node metastasis, as well as seven LMD adjacent normal samples. Tumour sample S1 was procured from the muscle invasive tumour (clinical stage T3b) and tumour samples S2–S4 were procured from three non-muscle invasive tumours (clinical stage Ta). Presented are all validated mutations. Furthermore, only mutations present in at least 5% of the tumour regions were included. LMD tumour regions were grouped using unsupervised hierarchical cluster analysis and clusters are indicated above the heat map. Data from normal samples were clustered independently (sample wise). Variants were classified as shared if present in more than 90% of the tumour samples. Oncogenes (red), tumour suppressor genes (blue), and IntOGen bladder cancer drivers (green) are annotated to the right of the heat map. Further, the Qiagen Clinical Insight Software was used to identify variants, for which therapeutics are available (T), in clinical trials (C_T_), or if the variant is pathogenic (P). Allele frequencies are presented ranging from >0 to 1 (an error rate of 1% was applied, hence if present, the variant is at least present at 1% in the sample). Grey indicates less than 4 alternate reads but an allele frequency greater than or equal to 1%, and could hence potentially be present if read depth was higher. Light blue indicates a failed amplicon. Dark blue equals no mutation. Lower heat map: transcriptomic profiling of corresponding regions presented in the upper heat map. Presented are normalized Ct values of genes of the following gene classes: luminal, differentiation, basal, high progression risk, and low progression risk. ND (grey): not determined. Missing value (white): sample has not been profiled. (**b**) Normal sample analysis: targeted sequencing of normal samples with a less stringent error correction applied (0.5%). The allele frequencies are presented on a different scale from present (>0) to 5%. (**c**) Illustration of sampling from cystectomy: see Supplementary Figure S2 for detailed overview of LMD regions.

Patient 1 presented with a T3b tumour (named S1 in Fig. 1a), three Ta tumours (named S2, S3, and S4 in Fig. 1a), and a pelvic lymph node metastasis. We selected 207 mutations inferred from WES and performed targeted amplicon sequencing (mean read depth = 4250 reads) on 33 tumour DNA samples (including bulk biopsies) and 7 LMD samples from the adjacent urothelium. We identified a clonal origin for all analysed tumour regions (Fig. 1a). Cluster analysis defined three clusters with different mutational patterns. As expected based on histopathological features, cluster 1 (C1) comprised all regions originating from the T3b tumour (S1) and the lymph node metastasis; cluster 2 (C2) comprised all regions from one of the Ta tumours (S2) and cluster 3 (C3) comprised all regions from the two remaining Ta tumours (S3 and S4, Fig. 1a). Mutations in known driver genes (the tumour suppressor genes *BAP1*, *MLL2* (also known as *KMT2D*), *CREBBP*, *EP300*, and the oncogene *FGFR3*) were shared between all samples while additional mutations in other oncogenes (*DNMT1* and *ERBB2*) were found exclusively in the cluster containing muscle invasive tumour regions. Finally, an additional mutation in *MLL2* was found only in cluster C2. Transcriptional profiling classified the Ta tumours as luminal (high expression of luminal markers such as GATA3, PPARG, FOXA1 and XBP1 as well as differentiation markers such as UPK1A and KRT20) as well as non-aggressive (high expression of SKAP2, FABP4, MBNL2 and ID1). The muscle invasive tumour, however, showed a basal subtype (higher expression of CDH3, CD44, KRT5 and KRT6A) and likewise high aggressiveness (higher expression of KPNA2, BIRC5, CDC25B, COL4A1, and MSN).

Targeted amplicon sequencing of the adjacent urothelium showed that some mutations observed in the tumours were present at low frequencies in these samples (range: 1 to 18.8%, median: 2.1%). Especially shared mutations were frequently observed (Fig. 1a) in the normal urothelium compared to private mutations (p value < 0.001, Fischer’s exact test). Following, we applied a less stringent error correction (see Materials and methods) and additional mutations were identified in the adjacent urothelium (Fig. 1b) – especially mutations identified as shared in the tumours. Indeed, some mutations, including a mutation in *BAP1*, were present in all normal samples.

Accordingly, we observed a clonal relationship between all samples analysed, shared mutations were enriched in adjacent normal samples, and the different multifocal tumours had acquired several private mutations. Interestingly, the inter-tumour differences were reflected in different transcriptional subtypes with basal- and luminal-like characteristics.

#### Patient 2

**Figure 2:**
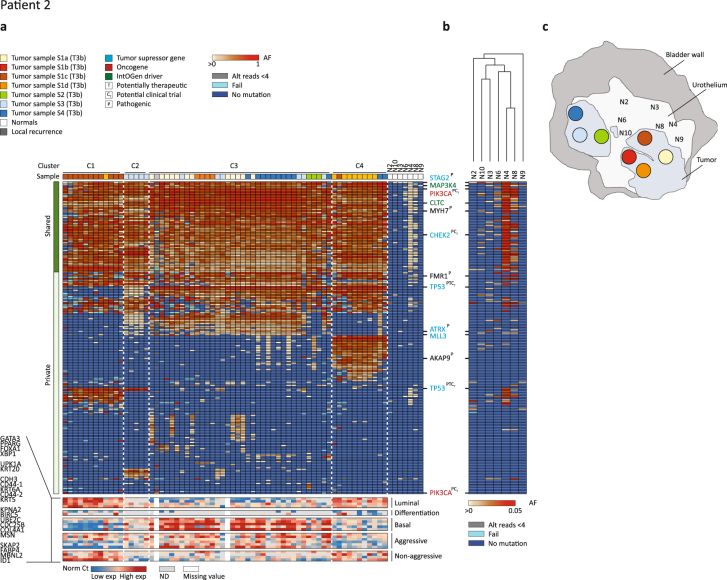
Detailed analysis of patient 2. Deep targeted sequencing was applied to 63 LMD tumour regions procured from multifocal tumour samples (4 samples from tumour 1 and one sample from each of tumour 2–4) as well as a sample from a local recurrence. Further, deep sequencing was applied to seven normal samples. The figure is annotated as Fig. 1. See Supplementary Figure S3 for detailed overview of LMD regions.

Patient 2 presented with multifocal and muscle invasive disease with large parts of the bladder being tumorous (Supplementary Fig. S3 and Fig. 2c). WES was performed on seven tumour samples (S1a, S1b, S1c, S1d, S2, S3, and S4) and on a local recurrence. We selected 538 mutations inferred from WES and performed targeted amplicon sequencing on 71 samples (including bulk samples) and 7 adjacent normal samples (mean read depth = 2567 reads). Here a clonal origin was also observed and cluster analysis revealed four main clusters indicative of four major subclones (Fig. 2a). Cluster 1 (C1) and cluster 4 (C4) were mainly comprised of regions from tumour samples S1c and S1d, respectively. Cluster 2 (C2) was comprised of regions from tumour sample S3 and cluster 3 (C3) was a mix of regions from tumour samples S1a, S1b, S2, S4, and the remaining regions from S3. Mutations in *STAG2*, *PIK3CA*, *CLTC*, and *CHEK2* appeared shared between all regions, whereas a mutation in *TP53* was shared between all regions except from C1 and appeared only subclonal in C2, as the frequencies were markedly lower in this cluster. However, an additional mutation in *TP53* was present only in cluster C1 and C2. *ATRX* and *MLL3* (also known as *KMT2C*) were mutated in all regions in C3 except regions originating from tumour sample S2. Transcriptional profiling showed that samples in cluster C1 and C4 had luminal-like characteristics and expressed genes associated with low aggressiveness whereas samples in cluster C3 were basal-like and showed higher expression of genes associated with high aggressiveness. Accordingly, the local recurrence clustered within cluster C3 with more aggressive characteristics.

Targeted sequencing of adjacent normal samples revealed that some of the mutations observed in the tumours were present at low frequencies (range: 1 to 31%, median: 3.3%; Fig. 2a). Again, mutations identified as shared in the tumour were significantly more often identified in the normal samples compared to private mutations (p value < 0.001, Fisher’s exact test). Applying less stringent error correction to data from normal samples showed additional mutations identified as shared in the tumours. No mutations were identified in all normal samples in this patient.

Consequently, we observed a clonal relationship of all tumour regions and the subclones observed were not directly associated with physical location in the bladder. Also in this case, mutations identified as shared in the tumours were enriched in the surrounding normal samples, and differences at the genomic level were reflected at the transcriptomic level.

#### Patient 3 and patient 4

**Figure 3:**
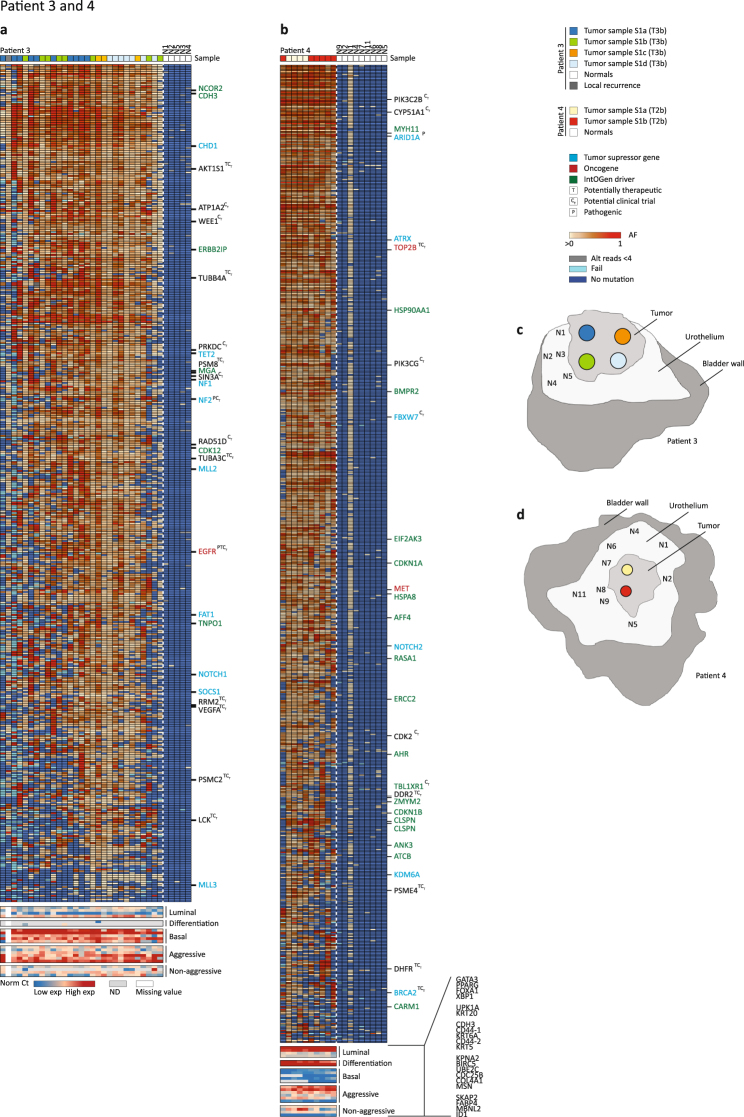
Detailed analysis of patient 3 and patient 4. (**a**) Patient 3. Deep targeted sequencing was applied to 28 LMD regions from four tumour samples taken from one large unifocal tumour, a sample from a local recurrence, as well as five normal samples. (**b**) Patient 4. Deep targeted sequencing was applied to 10 LMD regions from two tumour samples from a unifocal tumour as well as nine normal samples. (**c**) Illustration of sampling from cystectomy for patient 3: see Supplementary Figure S4 for detailed overview of LMD regions. (**d**) Illustration of sampling from cystectomy for patient 4: see Supplementary Figure S5 for detailed overview of LMD regions. The figure is annotated as Fig. 1.

Patients 3 and 4 presented with unifocal tumours, and cluster analysis did not separate samples into major subclones in these two patients (Fig. 3a,b). Both patients had tumours with numerous mutations in known driver genes. The lack of genomic ITH was further reflected at the transcriptomic level where patient 3 showed a basal-like subtype and high aggressiveness in all regions from all four tumour biopsies and patient 4 showed a non-aggressive luminal-like subtype in all regions from both tumour biopsies. Few mutations were observed in the adjacent normal samples from these patients except from sample N1 in patient 4, which showed presence of most of the tumour specific mutations (Fig. 3a,b). Applying less stringent error correction did not reveal presence additional tumour specific mutations in the normal samples (data not shown).

Consequently, a clonal relationship was observed for all samples analysed, but no major subclones were observed at the genomic or transcriptomic level. Hence the two patients presented with low spatial ITH, and the adjacent urothelium showed little evidence of field disease (apart from sample N1 in patient 4), which reflects the unifocal disease presentation observed in these two patients.

### Identification of potential therapeutic targets

Finally, we annotated possible therapeutic targets in order to retrospectively look into the possibilities for administering “precision medicine”. Potential therapeutic targets (with FDA approved drugs available or drugs in clinical trials) were identified and a full list can be seen in Supplementary Table S3. A shared *FGFR3* activating mutation was found in patient 1 and could potentially be targeted by FGFR inhibitors. Another mutation in *ERBB2* found in the muscle invasive tumour (S1) could be a treatment target for the more aggressive subclone. For patient 2, mutations in *PIK3CA* and *CHEK2* were identified in all tumour regions and may be potential therapeutic targets (drugs are still being tested in clinical trials). In patients 3 and 4, numerous mutations were potential therapeutic targets, though only few were identified in all tumour regions. In the case of patient 3, *AKT1S1* was altered in all regions but one, indicating possible benefit of Everolimus. Finally, in patient 4, a mutation in *TOP2B* identified in all tumour regions could indicate that the patient would benefit from treatment with a topoisomerase inhibitor and another mutation in *HSP90AA1* (also identified in all tumour regions) could possibly lead to a better response to anthracyclines and cisplatin.

## Discussion

Here we report a comprehensive multi-regional analysis and an in-depth characterization of the heterogeneity in advanced bladder cancer in four patients. The genomic ITH was reflected at the transcriptomic level, revealing intra-patient differences in luminal- and basal-like subtypes and aggressiveness signatures. In addition, we found that some mutations mainly observed as shared in the tumours from patients with multifocal disease were also observed at low frequencies in the adjacent urothelium. Previously, a comparison of genomic and transcriptomic data obtained from tumour subpopulations procured by FACS from a single patient has been reported^24^.

Our analysis showed a clonal origin for all tumour regions in four patients with unifocal and multifocal advanced bladder cancer. Furthermore, patients with multifocal disease showed presence of subclonal evolution (three to four subclones), while patients with unifocal disease showed no subclonal diversification. This is in concordance with earlier studies of bladder tumours, where paired tumours have been analysed and few subclones were identified^11, 19^ and where multi-regional sequencing was performed^9^. A study applying single cell sequencing of a single bladder tumour also showed the presence of few subclones^25^. Whether the study applying single cell sequencing would have revealed a higher number of subclones if single cells from multi-regional biopsies had been analysed is unknown. In the present approach, we performed targeted amplicon sequencing based on mutations identified from WES. Consequently, our analysis is limited to the most frequent subclones detected at the obtained sequencing depth, and we may underestimate the number of subclones present. Future studies of additional patients are needed where small regions or preferable single cells are sequenced using e.g. whole exome or whole genome approaches to decipher the ITH further.

The level of ITH showed large differences between the four patients. Tumours from all patients showed presence of both shared and private mutations in driver genes. Patient 2 showed the highest degree of ITH, as four clusters were observed, with mutations in known disease drivers being subclonal. Whether some mutations in disease driver genes tend to be significantly more shared or private requires analyses of a large cohort and can therefore not be assessed in our study. Patient 3 and 4, on the other hand, showed a lower degree of ITH and clustering did not reveal any demarcation of subclones. The study by Zhang *et al*. showed that patients with lung adenocarcinomas with poor outcome had high ITH^5^. Whether the level of ITH in bladder cancer may serve as a prognostic marker requires further studies of larger patient cohorts.

**Figure 4:**
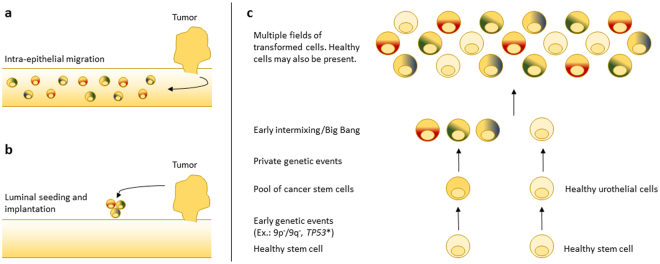
Models of field disease development in bladder cancer. (**a**) Intra-epithelial migration of multiple subclones gives rise to tumours of a clonal origin and presence of variants throughout the urothelium. (**b**) Luminal seeding followed by implantation gives rise to tumours of clonal origin. (**c**) Acquisition genetic alterations such as loss of 9p or 9q or mutation in *TP53* in an urothelial stem cell generates a pool of cancer stem cells of a clonal origin. Due to the increased proliferation, numerous mutations are acquired in an initial burst followed by intermixing and parallel expansion throughout the urothelium. This model gives rise to multiple fields.

Synchronous tumours in patient 1 showed acquisition of private mutations in concordance with a previous study^26^. Furthermore, the urothelium revealed presence of numerous mutations at low frequencies initially identified in the tumours. Hence, the mutations do not appear to be shared between all cells in the normal samples. This may be explained either by the presence of normal healthy cells or by a mix of different transformed cells. Of the mutations identified in the normal samples, a significantly higher number of the mutations were classified as shared rather than private in the tumours (Fig. 1). Previous studies have shown genomic alterations (mutations or loss of *RB1* or *TP53* as well as copy number alterations of chromosome 9, 13 and 17) in the normal urothelium in cystectomy specimens^15–17, 27–29^. These studies relied on technologies, which would not have detected mutations of low frequencies as in our analyses. We here suggest that the earlier studies identified early common events (e.g. loss of 9p or 9q or mutations in *TP53*) occurring in an urothelial stem cell and giving rise to a clonal population of cells, each acquiring a unique set of mutations followed by parallel expansion. This may result in multiple clonally related intermixed transformed fields. Tumours arising from a given field will harbour clonal variants mirroring the field from which it arose. The multiple field hypothesis (see Fig. 4c) explains the presence of low frequency mutations in the pre-neoplastic cells, as other fields are overlapping with different mutations. This theory is adapted from the big bang model in early stage colorectal cancer development^30^ and the theory of punctuated equilibrium^31^. The big bang model proposes that multiple molecular events takes place at an early time point, resulting in intermixed cellular subclones and similarly, the theory of punctuated equilibrium proposes punctuations or burst of genetic events intervened by stasis. A recent report applying single cell sequencing on triple negative breast cancers showed punctuated equilibrium in copy number evolution^32^. To confirm our hypothesis, deep WES of samples from the urothelium may be applied to reveal the presence of other field mutations not detected in the tumours. Another approach could be single cell sequencing including both copy number analysis and genome or exome sequencing as previously described^33^. Alternatively, the presence of low frequency mutations in the normal samples could also be derived from intra-epithelial migration (Fig. 4a), where carcinoma cells migrate in the urothelium giving rise to low frequent mutations due to presence of normal healthy cells, or from luminal seeding of carcinoma cells (Fig. 4b). However, intra-epithelial migration would require carcinoma cells to undergo epithelial-to-mesenchymal transition, and luminal seeding would require shredding of carcinoma cells, followed by implantation. Both events are considered molecularly complex^13^. Furthermore, to reflect the low frequencies of mutations that we observe in the normal urothelial samples, intra-epithelial migration and luminal seeding should be associated with little or no expansion of the tumour clones for the frequencies to remain low.

Gene expression subtypes have prognostic potential and may have clinical utility^34–37^. Here we showed that although different parts of a tumour are clonally related, ITH in molecular subtypes exists. Tumours of basal- and luminal-like subtypes have been proposed to arise from distinct uro-progenitors^38^. Our data may question this hypothesis as both molecular subtypes were identified within the same tumour (patient 2) and between clonally related tumours within a single patient (patient 1). Our data could indicate the presence of cell plasticity rather than intrinsic/inherited subtypes. Further analysis should be performed to investigate which molecular changes may mediate this possible subtype switch. Our assessment of molecular subtypes is based on selected genes defining basal and luminal subtypes only while many subtypes have been identified in MIBC^39^. ITH associated with molecular subtypes should be considered if e.g. basal- and luminal-like subtypes are used for administering therapeutic treatment in future clinical trials^40, 41^.

Aspects of personalized medicine were further assessed in light of ITH. The analysis was limited to mutations proposed targetable by the Qiagen Clinical Insight software (Supplementary Table S3). Targets identified in all analysed regions were identified in all four patients indicative of treatment with targeted therapies as well as chemotherapeutics. It is possible, that multiple pathways should be targeted simultaneously to obtain tumour regression as recently shown in a patient with anaplastic thyroid cancer^42^. Shared mutations in patients 1 and 2 could potentially be targeted to eradicate the underlying field disease – patient 1 presents with a shared *BAP1* mutation between the four tumours and *BAP1* is mutated in all samples from the urothelium. A recent report indicates that mesothelioma cells with inactivated *BAP1* are sensitive to EZH2 inhibition (epigenetic inhibitor)^43^. Patient 4 further showed a strong luminal-like subtype. Preliminary analysis has indicated a link between molecular subtypes of bladder cancer and response to PD-L1 inhibition, where patients with luminal tumours showed better response to PD-L1 inhibition^44^ pinpointing the importance of performing multi-regional subtyping. This study further sheds light on potential therapeutic targets in patients with advanced bladder cancer, and indicates possible treatment of pre-neoplastic cells surrounding the tumours.

## Methods

### Clinical samples

Patients included in the study where treated at Aarhus University Hospital in 2014. The patients underwent open radical cystectomy and extended lymph node dissection to the aortic bifurcation. All patients were cystectomised because of primary bladder cancer without neoadjuvant chemotherapy or radiation therapy. None of the patients had previously been diagnosed with bladder cancer. Biopsies from cystectomies were obtained, embedded in TissueTek OCT^TM^ Compound (Sakura, Finetek, Vaerloese, Denmark), snap frozen in liquid nitrogen, and placed at −80 °C. Two to seven biopsies were obtained from tumours from each patient along with six to 12 samples throughout the surrounding urothelium. Formalin Fixed and Paraffin Embedded (FFPE) specimens from a lymph node metastasis and local recurrences were obtained from Department of Pathology, Aarhus University Hospital. Blood samples were stored at −80 °C in EDTA tubes. All patients gave their informed written consent and the Danish National Committees on Heath Research Ethics (#1300174) approved the study and experimental protocols. The methods in the study were carried out in accordance with the approved guidelines and regulations. Detailed information on DNA and RNA extraction is described in Supplementary Data.

### Library construction and WES

WES was performed using either the Kapa Hyper Library Prep Kit (KapaBiosystems, Wilmington, MA, USA) for library construction followed by exome capture using Nimblegen SeqCap EZ Exome v3.0 Capture Kit (Roche) or the Nextera Rapid Capture Expanded Exome Kit (Illumina, San Diego, CA, USA). Input for the Kapa Hyper Library Prep was 1 µg of gDNA and input for the Nextera Rapid Capture was 50 ng of gDNA. Libraries were sequenced using the Illumina HiSeq 2000 or the NextSeq 500 platforms. WES was performed on bulk DNA from cross sections from each biopsy. The Kapa Hyper Library Prep kit was used for DNA procured from FFPE. When both library kits were used for a single patient, only overlapping genomic regions were investigated. For patient 1 libraries were constructed using pools of bulk DNA from cross sections (S1 + S2 and S3 + S4).

### Alignment, mapping, and variant calling

Sequence data were aligned, mapped, and variants called as previously described^11^ – see Supplementary Data. Furthermore, variants were sorted into categories based on the confidence of the call and tiers based on their estimated functional impact as previously described^11^. In short, categories were based on Mutect scores, presence of the alternate allele in the germline, and the frequency of the variant. The estimated impact on the encoded protein was derived using snpEff^45^ with tier 0 encompassing nonsense variants, tier 1 encompassing non-synonymous variants, and tier 2 encompassing synonymous variants.

### Targeted sequencing

Two TruSeq Custom Amplicon (TSCA) panels (Illumina) were designed for targeted re-sequencing of DNA from LMD regions based on variants identified from WES. We included all variants estimated to have functional impact by including all tier 0–1 variants with maximum one alternate allele detected in the germline to exclude sequencing errors (see Alignment, mapping, and variant calling). Additionally, if oncogenes or tumour suppressor genes^20^ were mutated, these were included in the amplicon panel disregarding functional impact estimation. This resulted in a small amplicon panel containing 207 mutations from patient 1 and 595 mutations from patient 3 and a large panel containing 538 mutations from patient 2 and 897 mutations from patient 4. TSCA libraries were constructed using the Illumina TruSeq Custom Amplicon v1.5 chemistry. Libraries were sequenced using the NextSeq 500 or the MiSeq platforms. Reads were stripped for adapters using ReadAdaptorTrimmer and mapped with BWA mem without further processing of the alignment.

### NGS error correction

Error rates for the targeted amplicon sequencing were calculated for each amplicon panel. As mutations identified from two patients were included in each amplicon panel, all amplicons designed for mutations in one patient were wildtype when investigating data for the other patient. For this analysis, we removed amplicons, which showed presence of the alternate allele in the blood and further removed the upper quartile of data from read depth. This was done, as few amplicons sometimes generate vast amounts of reads and could potentially be more error prone. We determined error rates for each type of nucleotide change and the highest rate was then multiplied by three. Analysis of samples from the surrounding urothelium was also investigated with the error rate multiplied by only 1.5.

### Fluidigm gene expression profiling

Expression profiling of RNA procured from LMD regions was performed using the Fluidigm 48.48 qPCR Dynamic Array microfluidic chips (Fluidigm/AH Diagnostics, Aarhus, Denmark). Detailed information is provided in Supplementary Data.

### Statistical analysis

Fischer’s exact test was used to test for statistical significance between shared and private mutations present in adjacent urothelium samples.

### Data availability

WES data is available via European Genome-phenome Archive (EGAC00001000145). All other data are available from the corresponding author upon request.

## Electronic supplementary material

Supplementary information
