# Supplementary material for: Comprehensive multiregional analysis of molecular heterogeneity in bladder cancer
Source: Sci Rep. 2017 Sep 15;7:11702. doi: 10.1038/s41598-017-11291-0 (PMC5600970; doi:10.1038/s41598-017-11291-0)
Supplement: Supplementary file 1 — Supplementary information [file 41598_2017_11291_MOESM1_ESM.pdf]

Comprehensive multiregional analysis of molecular heterogeneity in bladder cancer

Mathilde Borg Houlberg Thomsen[1], Iver Nordentoft[1], Philippe Lamy[1], Søren Vang[1], Line Reinert[2], Christophe Kamungu Mapendano[1], Søren Høyer[3], Torben F. Ørntoft[1], Jørgen Bjerggaard Jensen[4], and Lars Dyrskjøt[1*]

[1]Department of Molecular Medicine, Aarhus University Hospital, Palle Juul-Jensen Boulevard 99, 8200 Aarhus N, Denmark. [2]Department of Biomedicine, Aarhus University, 8000 Aarhus C, Denmark. [3]Department of Pathology, Aarhus University Hospital, Ndr. Ringgade, 8000 Aarhus C, Denmark. [4]Department of Urology, Aarhus University Hospital, Palle Juul-Jensen Boulevard 99, 8200 Aarhus N, Denmark.

Supplementary Data

DNA and RNA extraction

Genomic DNA was extracted from 5-10 cross sections of 10 µm for all tumor biopsies using the Gentra Puregene Tissue Kit (Qiagen, Copenhagen, Denmark), and four µm sections (top and bottom) where stained with haematoxylin and eosin. Only biopsies with a carcinoma cell percentage higher than 70% were included. An additional 15 sections of seven µm were cut for LMD and mounted onto Arcturus PEN membrane glass slides (Life Technologies, Naerum, Denmark) and stained with Histogene® LCM Frozen Section Staining Kit (Life Technologies) using manufacturers protocol. An additional four µm section was stained with haematoxylin and eosin stain for histological orientation. LMD followed by DNA extraction was performed from the first 9 of the 15 sections. LMD was performed from all 15 sections from normal biopsies where urothelium was scarce to ensure adequate

amounts of DNA. LMD followed by RNA extraction was performed on the 10th section from the tumor biopsies. All LMD procedures were performed by MBHT under supervision of LD. RNA extraction was carried out using the Qiazol lysis reagent (Qiagen) protocol with slight modifications. In brief, lysis was performed using 100 µl Qiazol for 30 min at room temperature followed by adding 900 µl Qiazol. Next, 200 µl chloroform (Merck, Darmstadt, Germany) was added followed by incubation for 5 min on ice and centrifugation for 5 min at 4 degrees at 12.000g. The aqueous phase was transferred to a low bind tube and 20 µg of Glycogen Carrier (Roche, Hvidovre, Denmark) was added. RNA was precipitated adding equal amount of isopropanol, thoroughly mixing and incubation at -20° C for one hour. RNA was pelleted by 15 min centrifugation at 4 degrees at 12.000g and washed twice in 70% ethanol. RNA was dissolved in RNase free water. For FFPE samples, macro dissections (applied on fine needle biopsies from local relapses) or punctures (for the lymph node metastasis) of a core size of 1.5 mm were performed to increase carcinoma cell purity. DNA from FFPE was extracted using QIAamp DNA FFPE Tissue Protocol with slight modifications. DNA concentrations were measured using either Quant-iT™ PicoGreen® DNA Assay Kit (Life Technologies) or the Qubit® dsDNA HS or BR Assay Kit (Life Technologies). RNA concentration and quality were assessed using the BioAnalyzer 2100 Pico Chip (Agilent Technologies, Santa Clara, CA, USA).

Fluidigm gene expression profiling

Total RNA was subjected to first strand cDNA synthesis using the SuperScript VILO cDNA Synthesis Kit (Thermo Fischer Scientific, Waltham, MA, USA) followed by pre-amplification of 1.25 µl of the generated cDNA using TaqMan PreAmp Master Mix (Fluidigm) and a 500nM pool of all assays. Pre-amplification was carried out at 10

min/95°C followed by 12 cycles of 15 sec/95°C and 4 min/60°C. An Exo I nuclease treatment (4U) was included by adding 2 µL to each pre-amplified reaction followed by 30 min digestion at 37°C and 15 min inactivation at 80 degrees (New England Biolabs, Ipswich, MA, USA). An overview of all assays can be found in Supplementary Table S3 (Sigma Aldrich, St. Louis, MI, USA). Assays were designed using Primer3plus software. Samples were diluted 1:5 prior to analysis. Next, all combined assays were diluted 1:5 and combined 1:1 with 2X Assay Loading Reagent (Fluidigm) resulting in concentrations of 500 nM in the final reaction. For all samples 4.5 µL cDNA was combined with 5.5 µl 1:10 mix of 20X DNA Binding Dye (Fluidigm) and 2X SsoFast EvaGreen Supermix with low ROX (BioRad, Copenhagen, Denmark). The dynamic array microfluidic chips were primed using injecting control line fluid (Fluidigm) and the Prime (113x) program on the IFC (Integrated Fluidic Circuit) Controller MX. Meanwhile, samples and assays where vortexed and centrifuged. Samples and assays (5 µL) where loaded onto sample and assay inlets and allowed to distribute running the Load Mix (113x) on an IFC Controller MX. The chip was then analyzed on the Biomark HD instrument immediately using EvaGreen single-probe assays and auto exposure. All samples were analyzed in either duplicates or triplicates. Melt-curve analysis was used to remove signal from non-target products and primer-dimers, and GAPDH was used as reference for normalization. The platform and method was initially validated and we found that normalized Ct values inversely correlated with RNAseq FPKM values (1) obtained from the same sample (R^2 = 0.60, 0.76 and 0.70, comparing to 1ng, 10 ng, and 100 ng RNA input for Fluidigm analysis respectively).

Assays used for Fluidigm expression analysis

Gene	Fwd	Rev	Tm Fwd	Tm Rev
FOXA1	ACTGTGAAGATGGAAGGGCA	CGCTCGTAGTCATGGTGTTC	64.7	63.4
EGFR	CTGGAGAAAGGAGAACGCC	CGGAACTTTGGGCGACTATC	63.9	65.6
CDH3	CACCAACCATCATCCCGACA	TCTGTGTTAGCCGCCTTCAG	69.2	65.4
CDKN1A	GGTGGACCTGGAGACTCTC	TGGGCGGATTAGGGCTTC	61.4	67.2
PKM	CTGGGCTGAGGACGTGGA	CCATCCGGTCAGCACAATG	67.9	67.7
SNAI2	CAAGGCGTTTTCCAGACCA	AGATTTGACCTGTCGCAAATG	66.7	63
FABP4	GGGCCAGGAATTTGACGAAG	TCCATCCCATTTCTGCACATG	67.7	68
COL4A1	CCACCAQTAGAGAGGAGCGAG	CTCATACAGACTTGGCAGCG	63.9	63.6
KRT20	TGTCCTGCAAATTGATAATGCT	AGACGTATTCCTCTCTCAGTCTCATA	63.2	62.3
CD44-2	CGGACACCATGGACAAGTTT	TGGAATACACCTGCAAAGCG	64.8	66
CD44-1	GCTGATCATCTTGGCATCCC	GCTCCATTGCCACTGTTGAT	66.8	65.1
TP63	TTTCTTAGCGAGGTTGGGC	ATGCCCTTCCAGATCGCAT	64.1	66.8
KDM1A	ATGTTGCTGCAGGATCATCT	TGGCTGGGTAGTTACGGATC	62.3	63.8
XBP1	TGTCACCCCTCCAGAACATC	TGGGTCCAAGTTGTCCAGAA	65.1	65.5
ID1	ACGACATGAACGGCTGTTACTCAC	CTCCAACTGAAGGTCCCTGATGTAG	68.2	67.8
GATA3	GTCCTGTGCGAACTGTCAGA	TTTTTCGGTTTCTGGTCTGG	60	60.1
BIRC5	GGTTGCGCTTTCCTTTCTGT	GCACTTTCTCCGCAGTTTCC	65.4	66
ERBB2	GAGGACCCCACAGTACCC	CTGGGGCCGAACATCTGG	61.9	69.1
CDC25B	TCATTTTCCACTGTGAATTTCTCATC	AGTCGTTGACAGCACGGTCTC	65	66.8
BCL2L1	GCAGGTATTGGTGAGTCGGA	CCACAAAAGTATCCCAGCCG	65	66.4
CCNE1	GGGGAGCTCAAAACTGAAGC	ACATGGCTTTCTTTGCTCGG	65	66.3
E2F3	GAGACTGAAACACACAGTCC	CCTGAGTTGGTTGAAGCC	57.1	60.9
UBE2C	ACCCAACATTGATAGTCCCTTG	CTGGTGACCTGCTTTGAGTAG	63.7	61.5
SKAP2	TGTGGGATTGTACTGGAGCT	ATTTCTCCTACCACCAGCC	62.2	64.1
UPK1A	CTCAACGAGGAGGGCTGC	AGATACCCCACGTGTAGCTG	66.3	61.6
PPARG	AGCCCAAGTTTGAGTTTGCT	GAGGACTCAGGGTGGTTCAG	62.7	63.8
THY1	CACCACTCTGGCCATTCC	CTCACACTTGACCAGTTTGTCTCT	64.4	63.5
MSN	AGGAGAAGCACCAGAAGCAG	CTTCAGCCTCTCCATCAGCT	63.7	63.7
MBNL2	CCCCAAAAGTTGTCAGGTTGA	GTGTGTCGGAGGGTGAAGAT	65.9	64.1
KPNA2	TGAAGGAATTGGCATGGTGG	GGCTTAGGGCAGGAGTC	69	62.9
AKT3	ACCTCAAGTAACATCTGACACAG	AAATTGAGGGAAATGCGGCC	59	68.9
TWIST1	AGTCTTACGAGGAGCTGCAG	ATCTTGCTCAGCTTGTCCGA	64.4	65.1
KRT5	GGAGCTCATGAACACCAAGC	TGGTCCAACTCCTTCTCCAC	64.8	64.2
c-MYC	ATTCTCTGCTCTCCTCGACG	TCCTCATCTTCTTGTTCCTCCT	63.7	63
FGFR3	GCACACACGACCTGTACATG	CACCCGGGGAGTACTGCT	62.9	65.2
SOX2	GCTCGCAGACCTACAGAAC	TGGAGTGGGAGGAAGAGGTA	62.5	63.6
CDKN2A	CACCAGAGGCAGTAACCATG	TGATGATCTAAGTTTCCCGAGGT	63.2	64.6
GAPDH	ATGGGGAAGGTGAAGGTCGG	GACGGTGCCATGGAATTTGC	69.3	69.7
UBC	GATTTGGGTGGCGGTTCTT	TGCCTTGACATTCTCGATGGT	66.1	66.8
ZEB1	GCATCCAAAGAGCAAGAAGC	ACTGGGCTGCTCAAGACTGT	63.9	64.1
STAG2	CTCAGAAGGGAGGATTGAGGA	GCTGGATCAAAGAAATCAGGCT	64.5	65.5
PROM1	ATTGGCATCTTCTATGGTTT	GCCTTGTCCTTGGTAGTGT	57.6	59.2
CCND1	CCTGTCCTACTACCGCCTCA	CACCTCCTCCTCCTCCTCTT	64.1	63.7
APOBEC3B	TTCGAGGCCAGGTGTATTTC	AGGGGGTCCAGGATACAAAC	60.1	60.1
EP400	GCCGGAAGCACAGTAGAGAC	TGCTGGAAAACTACCCCTTG	60	60.1
MALAT1	CCCACCCCCTTAATCAGACT	CAACAGCACAGCGGTACACT	60.2	60
NEAT1	GGGCCATCAGCTTTGAATAA	CTTGAAGCAAGGTTCCAAGC	60	60
KRT6A	AGTTTGCCTCCTTCATCGAC	CAGCAGGGTCCACTTTGTTT	59.3	60.1

Alignment, mapping, and variant calling

Performed as previously described (2) - see Supplementary Data for details. Raw reads from the sequencer were demultiplexed and converted to fastq-format using Illumina's bcl2fastq software allowing one mismatch in the barcode sequences.

Reads were quality controlled using fastQC and adapter trimming was conducted using the GATK (v3.1.1) ReadAdaptorTrimmer tool (3). Reads were mapped to the hg19 reference genome using BWA mem (v0.7.5a) (4). Optical and PCR-duplicates generated in the library construction were removed using Picard MarkDuplicates (v1.96) and the final alignments were realigned near indels using GATK IndelRealigner and base quality scores were recalibrated and corrected for technical artifacts using GATK BaseRecalibrator. Somatic mutations were called using Mutect (v1.1.4) (5).

Supplementary References

1. Hedegaard J, Lamy P, Nordentoft I, Algaba F, Hoyer S, Ulhoi BP*, et al.* Comprehensive Transcriptional Analysis of Early-Stage Urothelial Carcinoma. Cancer cell **2016**;30:27-42
2. Lamy P, Nordentoft I, Birkenkamp-Demtroder K, Thomsen MB, Villesen P, Vang S*, et al.* Paired Exome Analysis Reveals Clonal Evolution and Potential Therapeutic Targets in Urothelial Carcinoma. Cancer research **2016**;76:5894-906
3. DePristo MA, Banks E, Poplin R, Garimella KV, Maguire JR, Hartl C*, et al.* A framework for variation discovery and genotyping using next-generation DNA sequencing data. Nature genetics **2011**;43:491-8
4. Li H, Durbin R. Fast and accurate short read alignment with Burrows-Wheeler transform. Bioinformatics (Oxford, England) **2009**;25:1754-60
5. Cibulskis K, Lawrence MS, Carter SL, Sivachenko A, Jaffe D, Sougnez C*, et al.* Sensitive detection of somatic point mutations in impure and heterogeneous cancer samples. Nature biotechnology **2013**;31:213-9

Supplementary Figure S1

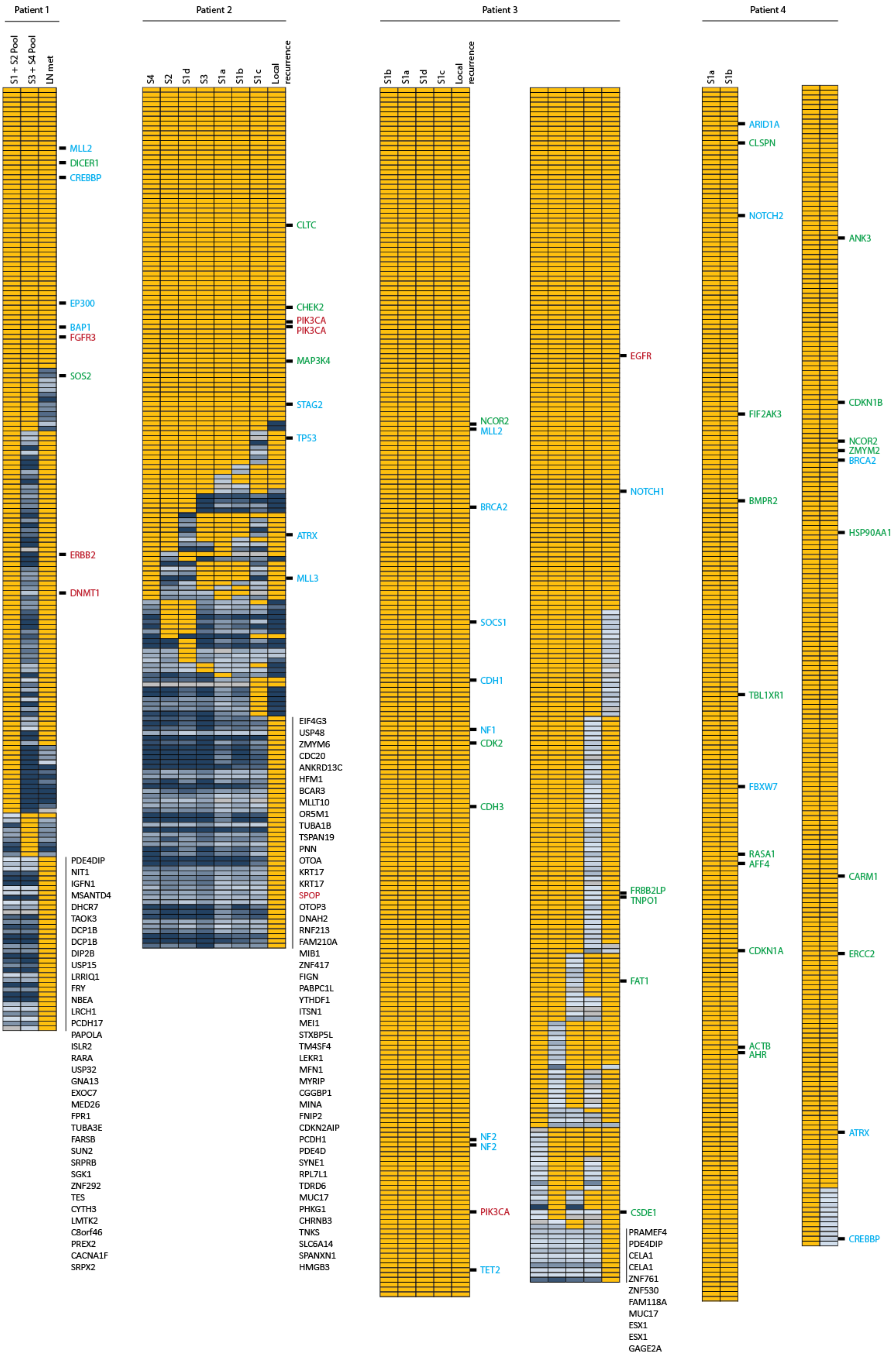

Supplementary Figure S2

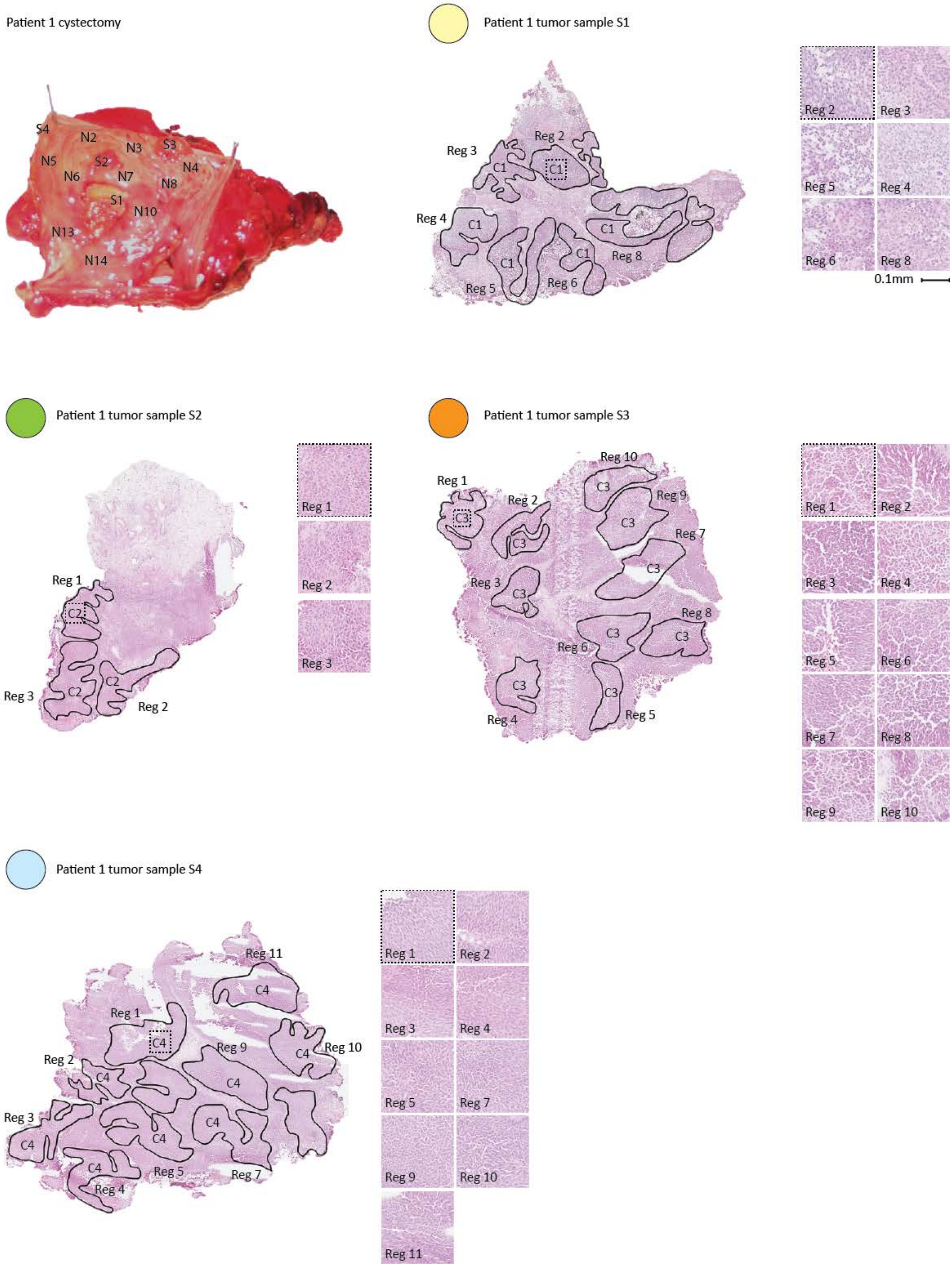

Supplementary Figure S3

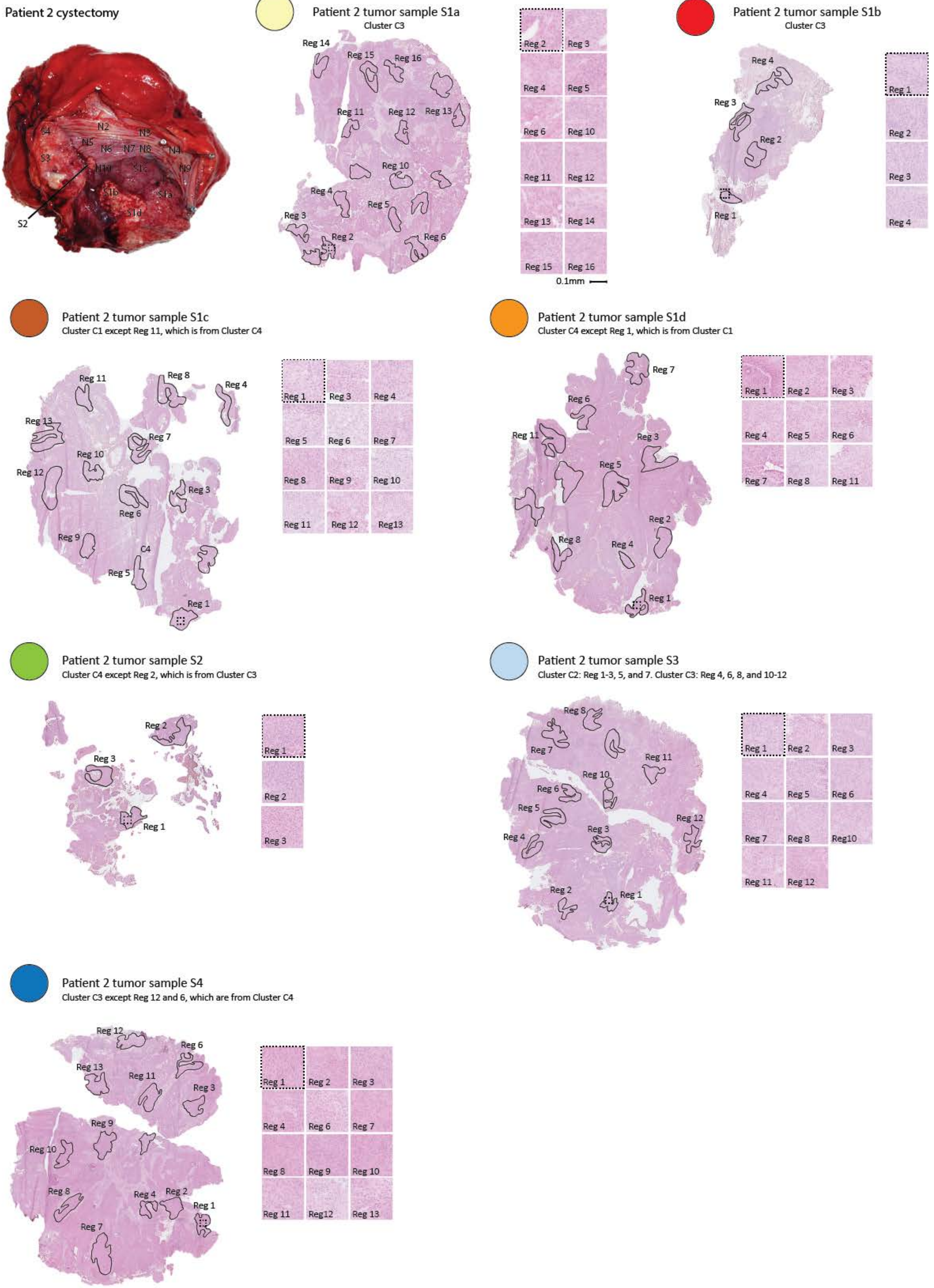

Supplementary Figure S4

Patient 3 cystectomy

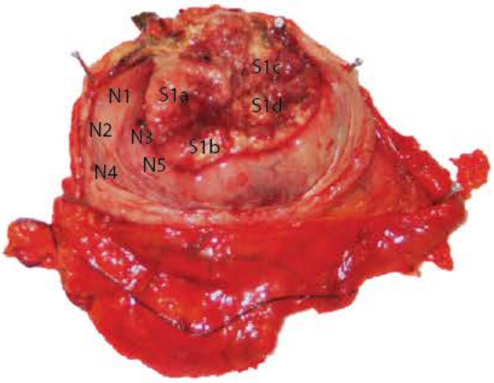

Patient 3 tumor sample S1a

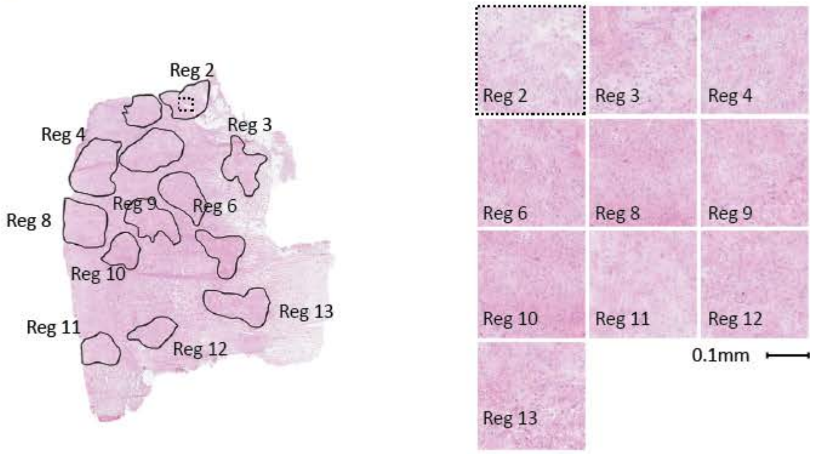

Patient 3 tumor sample S1b

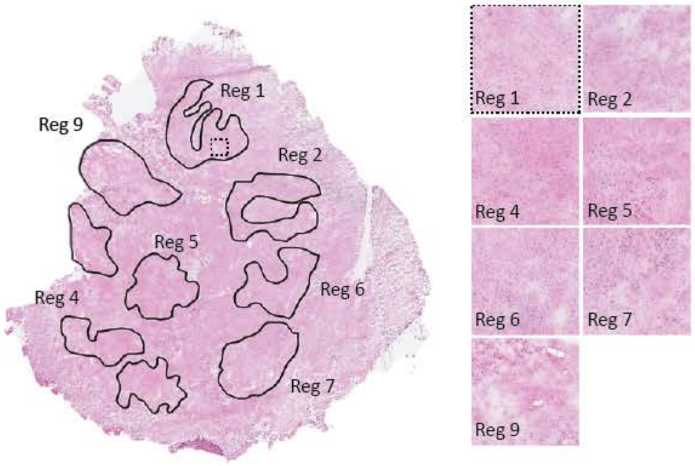

Patient 3 tumor sample S1c

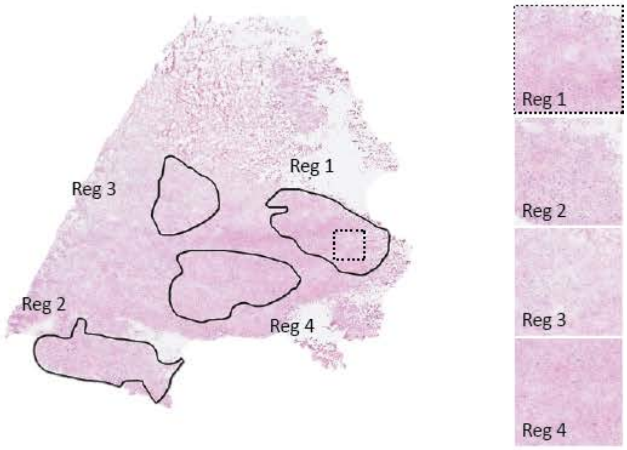

Patient 3 tumor sample S1d

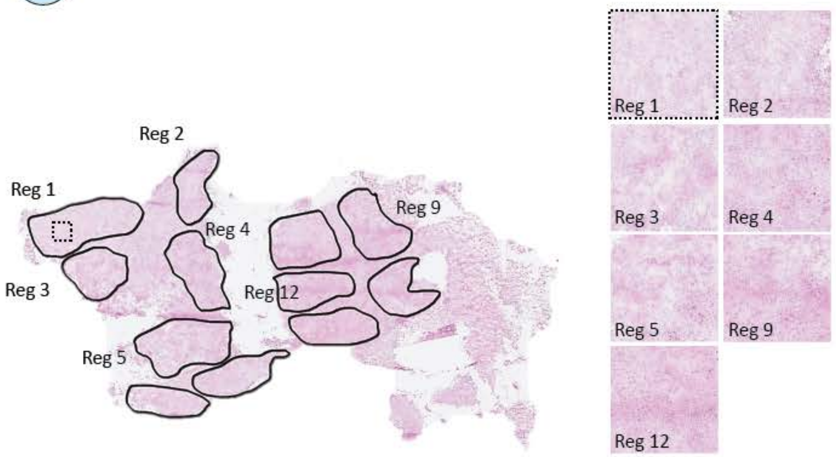

Supplementary Figure S5

Patient 4 cystectomy

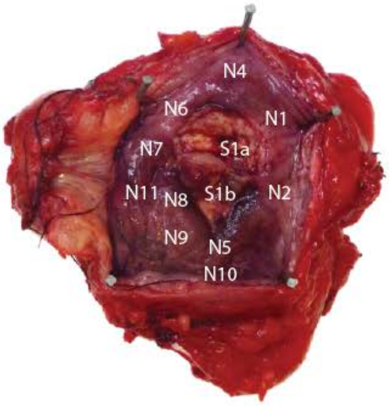

Patient 4 tumor biopsy S1a

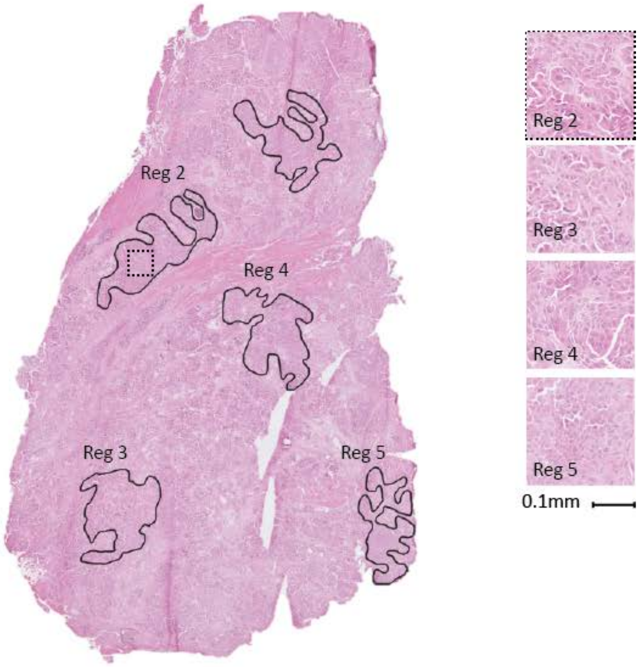

Patient 4 tumor biopsy S1b

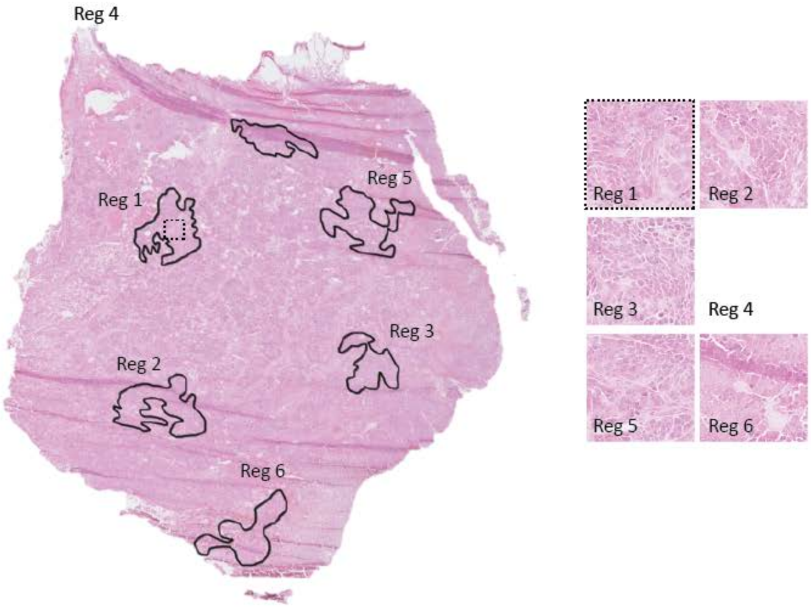

Supplementary figure legends

Supplementary Figure S1: Multi-regional WES of advanced bladder tumours and paired lymph node metastasis or local recurrences. Heat maps presenting WES data from multiple tumour samples from patient 1-4 together with lymph node metastasis for patient 1 and local relapse samples in patient 2 and 3. The data is presented dichotomized with yellow being present (called with a category score of 1 or 2 in least one sample). Only mutations with an estimated functional impact are presented. Blue indicates that the mutation is not present with the degree of blue showing the number of obtained reads (range: 10-1048 reads, grey indicates less than 10 reads). To identify potential driver mutations, gene names are annotated for acquired mutations in the lymph node metastasis in patient 1 and the local recurrence samples for patient 2 and 3 to the right of the respective heat maps. Further, known oncogenes (red), tumour suppressor genes (blue), and IntOGen bladder cancer drivers (green) are annotated to the right of each heat map. For patient 1, an exome was performed on a pool of DNA from bulk samples S1 and S2 as well as a pool of DNA from bulk samples S3 and S4. LN Met: Lymph node metastasis.

Supplementary Figure S2: Histologic overview of laser micro-dissection for patient 1. For each sample, multiple regions were procured for DNA (nine tissue sections) and RNA (one tissue section) extraction. Images taken at 40x magnifications are shown to the left of each sample. Genomic clusters presented in Figure 1A are indicated for each profiled region. The colour indicates sampling as presented in Figure 1C. Regions procured, but not analysed, are not annotated. Sample S1: six regions from a muscle invasive tumour (T3b) profiled. Sample S2:

three regions from a Ta tumour profiled. Sample S3: ten regions from a Ta tumour profiled. Sample S4: nine regions from a Ta tumour profiled.

Supplementary Figure S3: Histologic overview of laser micro-dissection for patient 2. For each sample, multiple regions were procured for DNA (nine tissue sections) and RNA (one tissue section) extraction. Images taken at 40x magnifications are shown to the left of each sample. Genomic clusters presented in Figure 2A are indicated for each profiled region. The colour indicates sampling as presented in Figure 2C. Regions procured, but not analysed are not annotated. Four samples were sampled from a T3b tumour (Sample S1a-S1d) and additional three samples were samples from three adjacent tumours, also muscle invasive. Sample S1a: 12 regions profiled. Sample S1b: four regions profiled. Sample S1c: 12 regions profiled. Sample S1d: nine regions profiled. Sample S2: three regions profiled. Sample S3: 11 regions profiled. Sample S4: 12 regions profiled.

Supplementary Figure S4: Histologic overview of laser micro-dissection for patient 3. For each sample, multiple regions were procured for DNA (nine tissue sections) and RNA (one tissue section) extraction. Images taken at 40x magnifications are shown to the left of each sample. The colour indicates sampling as presented in Figure 3C. Regions procured, but not analysed, are not annotated. The four samples were sampled from a T3b tumour. Sample S1a: ten regions profiled. Sample S1b: seven regions profiled. Sample S1c: four regions profiled. Sample S1d: seven regions profiled.

Supplementary Figure S5: Histologic overview of laser micro-dissection for patient 4. For each sample, multiple regions were procured for DNA (nine tissue sections) and RNA (one tissue section) extraction. Images taken at 40x

magnifications are shown to the left of each sample. The colour indicates sampling as presented in Figure 3D. Regions procured, but not profiled, are not annotated. Two samples were sampled from a T2b tumour (Sample S1a-S1b). Sample S1a: four regions profiled. Sample S1b: six regions profiled.

Supplementary tables and table legends

Supplementary Table S1: Clinical and pathological information.

Patient:	Patient 1	Patient 2	Patient 3	Patient 4
Gender	Female	Female	Male	Male
Age at Diagnosis	70	69	73	86
T Stage (Clinical)	3b	3b	3b	2b
Grade	High	High	High	High
N status	1	0	0	0
Focality	Multifocal	Multifocal	Unifocal	Unifocal
Treatment	*Cystectomy + Lymphadenoectomy*	*Cystectomy + Lymphadenoectomy*	*Cystectomy + Lymphadenoectomy*	*Cystectomy + Lymphadenoectomy*
Neo adj. Chemotherapy	No	No	No	No
Follow Up (Days)	4	506	279	613
Relapse	No	Yes (Vagina)	Yes (Pelvis)	No
Adj. Chemotherapy	-	Gemcitabine/ Cisplatin	No	-
Time to Relapse	-	218	142	-
Died of Disease	Yes[a]	Yes	Yes	No

[a] Patient 1 died of complication following surgery

Supplementary Table S2: Overview of WES and targeted sequencing.

Samples	Patient 1	Patient 2	Patient 3	Patient 4
Germline DNA	Leucocytes	Leucocytes	Leucocytes	Leucocytes
Samples from Adjacent Urothelium	7	7	5	9
Tumor Biopsies (T)	4 (T1-T4)[a]	7 (T1a-T1d, T2-T4)	4 (T1a-T1d)	2 (T1a-T1b)
Lymph Node Metastasis (N)	1	No	No	No
Recurrence (M)	No	Local	Local	No
Type (T/N/M)[b]	FF/FFPE/-	FF/-/FFPE	FF/-/FFPE	FF/-/-

Whole Exome Sequencing	Patient 1	Patient 2	Patient 3	Patient 4
Number of Exomes	3*	8	5	3
Bait Set	Nextera	SeqCap_EZ	Nextera	SeqCap_EZ
Mean Target Coverage (Tumor)	86-107X	31-82X	52-113X	62-65X
Mean Target Coverage (Germline)	109X	55X	52X	72X
Total Mutations[c]	452	900	961	1343
Tier 0 Mutations	24	50	47	72
Tier 1 Mutations	273	652	651	817
Tier 2 Mutations	155	198	263	454
**Targeted Sequencing**	**Patient 1**	**Patient 3**	**Patient 2**	**Patient 4**
Re-sequenced Mutations[d]	207	538	595	897
Mean Read Depth (Tumor)	4250	2567	4273	2712
Mean Read Depth (Germline)	2061	5541	5302	4937

[a]T1+T2 was pooled in one exome and T3+T4 was pooled in another exome, another exome was run on the lymph node metastasis

[b]All FFPE samples were sequenced using SeqCap_EZ protocol

[c]Tier 0: High impact, Tier 1: Moderate impact, Tier 2: Low impact

[d]Panels include all tier 0 and 1 mutations along with selected driver genes

Supplementary Table S3: Potential therapies identified using Qiagen Clinical Insight Software. For each patient the following is listed: Gene, chromosomal variation, protein variation, Significance called by Qiagen Clinical Insight, type of mutation, the proposed activity, and potential drugs already approved by the FDA, and the number of potential clinical trials. Further, genes are coloured as oncogenes (red), tumour suppressor genes (blue), and IntOGen bladder cancer drivers (green).

Patient 1 Genes	Chromosomal Variation	Protein Variation	Significance	Type	Activity	Clonality	Drugs	Clinical Trials
FGFR3	c.1118A>G	p.Y373C	Likely Pathogenic (Bladder Cancer)	Missense	Gain	Shared	Ponatinib, Nintedanib, Pazopanib, Lenvatinib, Reforabenib	139
ABCC1	c.175C>T	p.R59*	Uncertain Significance (Cancer)	Stop Gain	Loss	Private		1
DNMT1	c.3212G>A	p.C1071Y	Uncertain Significance (Cancer)	Missense	Normal	Private		83
ERBB2	c.829G>T	p.D277Y	Uncertain Significance (Cancer)	Missense	Normal	Private	Afatinib, Trastuzumab, Osimertinib, Bosutinib, Erlotinib	127
FANCD2	c.3339G>C	p.Q1113H	Uncertain Significance (Bladder Cancer)	Missense	Normal	Private		2
PIK3C3	c.2368A>G	p.T790A	Uncertain Significance (Cancer)	Missense	Normal	Private		1
**Patient 2 Genes**	**Chromosomal Variation**	**Protein Variation**	**Significance**	**Type**	**Activity**		**Drugs**	**Clinical Trials**
CHEK2	c.958A>T	p.K320*	Pathogenic (Bladder Cancer)	Stop Gain	Loss	Shared		2
PIK3CA	c.1633G>A	p.E545K	Pathogenic (Bladder Cancer)	Missense	Gain	Shared		82
TP53	c.839G>C	p.R280T	Pathogenic (Bladder Cancer)	Missense	Loss	Private	Lenalidomide, Chlorambucil/Obinutuzumab, Alemtuzumab, Idelalisib, Cyclophosphamide/Fludarabine phosphate/Rituximab, Idelalisib/Rituximab, Lenalidomide/Rituximab, Cyclophosphamide/Fludarabine, Cytarabine/Fludarabine phosphate/Oxaliplatin/Rituximab, Ibrutinib, Fludarabine phosphate/Rituximab, Ofatumumab, Venetoclax, Methylprednisolone/Rituximab, Alemtuzumab/Rituximab	19
**Patient 3 Genes**	**Chromosomal Variation**	**Protein Variation**	**Significance**	**Type**	**Activity**		**Drugs**	**Clinical Trials**
NF2	c.1063G>T	p.E355*	Pathogenic (Cancer)	Stop Gain	Loss	Private		4
NF2	c.172G>A	p.E58K	Uncertain Significance (Cancer)	Missense	Loss	Private		4
AKT1S1	c.683G>C	p.R228P	Uncertain Significance (Cancer)	Missense	Normal	Shared	Everolimus	61
ATP1A2	p.E477D	c.1431G>C	Uncertain Significance (Cancer)	Missense	Normal	Shared		4
EGFR	c.1701G>T	p.M567I	Uncertain Significance (Bladder Cancer)	Missense	Normal	Private	Afatinib, Afatinib/Cetuximab, Alectinib, Cetuximab/Cisplatin/Vinorelbine, Gefitinib, Crizotinib, Erlotinib, Ceritinib, Carboplatin/Paclitaxel, Carboplatin/Erlotinib/Paclitaxel, Cetuximab, Lapatinib, Panitumumab, Bosutinib, Necitumumab, Vandetanib	240
LCK	c.256G>A	p.E86K	Uncertain Significance (Cancer)	Missense	Loss	Private	Ponatinib, Vandetanib, Bosutinib, Pazopanib, Dasatinib	99
PRKDC	c.4791G>C	p.L1597F	Uncertain Significance (Bladder Cancer)	Missense	Loss	Private		2
PSMA8	c.753A>C	p.K251N	Uncertain Significance (Cancer)	Missense	Normal	Private	Carfilzomib, Bortezomib	74
PSMC2	c.1191C>G	p.I397M	Uncertain Significance (Cancer)	Missense	Loss	Private	Carfilzomib, Bortezomib	74
RAD51D	c.233C>G	p.S78C	Uncertain Significance (Bladder Cancer)	Missense	Loss	Private		1
RRM2	c.166C>G	p.R56G	Uncertain Significance (Bladder Cancer)	Missense	Loss	Private	Ludarabine phosphate, Cladribine, Gemcitabine	231
SIN3A	c.1495G>C	p.E499Q	Uncertain Significance (Bladder Cancer)	Missense	Loss	Private		1
TUBA3C	c.454C>G	p.L152V	Uncertain Significance (Bladder Cancer)	Missense	Loss	Private	Cabazitaxel, Brentuximab Vedotin, Vincristine, Vinorelbine, Vinblastine, Vinflunine, Ixabepilone, Trastuzumab Emtansine, Eribulin, Paclitaxel	459
TUBB4A	c.211G>A	p.G71S	Uncertain Significance (Bladder Cancer)	Missense	Normal	Private	Trastuzumab Emtansine, Eribulin, Vinblastine, Vinfluine, Vincristine, Docetaxel, Paclitaxel, Brentuximab Vedotin, Carbazitaxel, Vinorelbine	459
VEGFA	c.744G>C	p.E248D	Uncertain Significance (Bladder Cancer)	Missense	Normal	Private	Aflibercept, Bevacizumab	118
WEE1	c.1426G>A	p.E476K	Uncertain Significance (Cancer)	Missense	Loss	Shared		12
**Patient 4 Genes**	**Chromosomal Variation**	**Protein Variation**	**Significance**	**Type**	**Activity**		**Drugs**	**Clinical Trials**
ARID1A	c.1435C>T	p.Q479*	Pathogenic (Bladder Cancer)	Stop Gain	Loss	Shared		-
BRCA2	c.9392C>T	p.S3131F	Uncertain Significance (Bladder Cancer)	Missense	Normal	Private	Olaparib, Platinum agent, Tamoxifen, Antrhacycline/Chemotherapy, Cisplatin/Gemcitabine, Platinum Chemotherapy	42
CDK2	c.158C>T	p.S53F	Uncertain Significance (Cancer)	Missense	Loss	Private		4
CYP51A1	c.614C>T	p.S205F	Uncertain Significance (Cancer)	Missense	Loss	Shared		9
DDR2	c.59C>G	p.S20C	Uncertain Significance (Cancer)	Missense	Normal	Private	Regorafenib, Imatinib	56
DHFR	c.110G>A	p.R37K	Uncertain Significance (Bladder Cancer)	Missense	Normal	Private	Permetrexed, Methotrexate, Pralatrexate	57
FBXW7	c.1637C>T	p.S546L	Uncertain Significance (Bladder Cancer)	Missense	Normal	Shared		1
HSP90AA1	c.433C>T	p.Q145*	Uncertain Significance (Bladder Cancer)	Stop Gain	Loss	Shared	Antrhacycline, Cisplatin	171
PIK3C2B	c.3806G>T	p.R1269L	Uncertain Significance (Cancer)	Missense	Loss	Shared		50
PIK3CG	c.2263G>A	p.E755K	Uncertain Significance (Bladder Cancer)	Missense	Normal	Shared		64
PSME4	c.3571G>A	p.E1191K	Uncertain Significance (Cancer)	Missense	Normal	Private	Carfilzomib, Bortezomib	62
TBL1XR1	c.31T>G	p.L11V	Uncertain Significance (Bladder Cancer)	Missense	Loss	Private		1
TOP2B	c.4748C>T	p.S1583L	Uncertain Significance (Bladder Cancer)	Missense	Normal	Shared	Etoposide phosphate, Valrubicin, Etoposide, Doxorubicin, Irinotecan, Pixantrone, Daunrubicin, Teniposide, Epirubicin	210

[a]Normal: Variant unknown, expected to be normal, [b]Loss: Loss of function, [c]Gain: Gain of function, [d]Clinical Trials: Numbers of recruiting clinical trials based on all cancers
